# Coronary Calcium Is Elevated in Patients with Myocardial Infarction without Standard Modifiable Risk Factors

**DOI:** 10.3390/jcm13092569

**Published:** 2024-04-27

**Authors:** Jeffrey L. Anderson, Stacey Knight, Li Dong, Heidi T. May, Viet T. Le, Tami L. Bair, Kirk U. Knowlton

**Affiliations:** 1 Intermountain Medical Center Heart Institute, Salt Lake City, UT 84107, USA; stacey.knight@imail.org (S.K.); li.dong@imail.org (L.D.); heidi.may@imail.org (H.T.M.); viet.le@imail.org (V.T.L.); tami.bair@imail.org (T.L.B.); kirk.knowlton@imail.org (K.U.K.); 2 Department of Internal Medicine, University of Utah School of Medicine, Salt Lake City, UT 84112, USA; 3 Rocky Mountain University of Health Professions, Provo, UT 84606, USA

**Keywords:** coronary artery calcium, myocardial infarction, risk factors, statin

## Abstract

**Objectives**: Recent reports have highlighted myocardial infarction (MI) patients without standard modifiable risk factors (SMRF), noting them to be surprisingly common and to have a substantial risk of adverse outcomes. The objective of this study was to address the challenge of identifying at-risk patients without SMRF and providing preventive therapy. **Methods**: Patients presenting between 2001 and 2021 to Intermountain Health catheterization laboratories with a diagnosis of MI were included if they also had a coronary artery calcium (CAC) scan by computed tomography within 2 years. SMRF were defined as a clinical diagnosis or treatment of hypertension, hyperlipidemia, diabetes, or smoking. The co-primary endpoints in SMRF-less patients were: (1) proportion of patients with an elevated (>50%ile) CAC score, and (2) an indication for statin therapy (i.e., CAC ≥ 100 AU or ≥75%ile). The 60-day and long-term major adverse cardiovascular events were determined. A comparison set included MI patients with SMRF. **Results**: We identified 429 MI patients with a concurrent CAC scan, of which 60 had no SMRF. SMRF status did not distinguish most risk factors or interventions. No-SMRF patients had a high CAC prevalence and percentile (82% ≥ 50%ile; median, 80%ile), and 77% met criteria for preventive therapy. As expected, patients with SMRF had high CAC scores and percentiles. Outcomes were more favorable for No-SMRF status and for lower CAC scores. **Conclusions**: Patients without SMRF presenting with an MI have a high prevalence and percentile of CAC. Wider application of CAC scans, including in those without SMRF, is promising as a method to identify an additional at-risk population for MI and to provide primary preventive therapy.

## 1. Introduction

Contemporary reports, mostly from outside of the United States, have highlighted myocardial infarction (MI) patients without standard modifiable risk factors (SMRF), designated in several earlier reports as SMuRF-less, and have noted them to be surprisingly common (i.e., 14–27% of ST-elevation MI [STEMI] presentations, 11–22% of non-STEMI [NSTEMI] presentations) and to have a significant risk of adverse outcomes [1,2,3,4,5,6,7,8,9,10].

Recently, we have reported on the frequency and outcomes of MI patients, both with STEMI [6] and with NSTEMI [10], who present without SMRF in a large United States healthcare system. We confirmed that No-SMRF status is frequently present in our system (26% of STEMI, 18% of NSTEMI presentations). Adverse outcomes were common although, in contrast to some earlier and non-US reports [2,3,4,5,8,9], early prognosis was similar (STEMI) or better (NSTEMI) than in patients with SMRF, and long-term prognosis was improved [6,10].

These reports raise the question of how these patients with no SMRF can be identified in advance of their coronary event and be provided with effective preventive care (e.g., statins, aspirin) for their atherosclerotic cardiovascular disease (ASCVD). Several non-traditional risk factors could underlie a predisposition to ASCVD in SMRF-less individuals. These include subthreshold elevations of traditional risk factors or non-standard lifestyle and psychosocial measures. Biomarker evidence of subclinical ASCVD should also be considered, such as cardiac troponin. For example, recent investigations suggest that serum cardiac troponin concentrations are higher in people with subclinical coronary artery disease (CAD) than in those without atherosclerosis [11]. Investigational applications of polygenetic risk scores have also shown promise in identifying individuals at high ASCVD risk.

This study focused on the potential utility of coronary artery calcium (CAC) determinations as an approach to identifying SMRF-less individuals with subclinical CAD at risk for myocardial infarction (MI) [12,13,14]. The presence of CAC has been shown to be a measure of coronary atherosclerosis burden and a potent prognosticator for the risk of future coronary events [12]. As coronary plaques evolve, calcium is deposited as part of the injury–repair cycle. These advanced plaques are prone to rupture, provoking coronary thrombosis and clinical events. Coronary calcium, once present, generally does not regress with lipid-lowering therapy, but it remains fixed and increases slowly over time along a continuous percentile curve specific for age and sex. Current guidelines recommend CAC scoring for risk refinement in selective patients, i.e., those in whom standard risk-factor assessment is ambiguous, but not universally, and not in otherwise low-risk individuals by standard risk-factor assessment [13].

## 2. Materials and Methods

*Study Aims and Institutional Review Board Approval:* The co-primary aims of this retrospective database study were: (1) to determine the proportion of patients without SMRF with an elevated (>50 percentile) coronary artery calcium (CAC) score (using the MutiEthnic Study of Atherosclerosis [MESA] criteria) [12], and (2) the presence of an indication for statin therapy with or without aspirin (i.e., CAC score ≥ 100-Agatston Units [AU] and/or ≥75%ile) [13,14]. The 60-day and long-term major adverse cardiovascular event (MACE) outcomes were determined. For comparison, we also assessed the results for a parallel set of MI patients with 1–4 SMRF. This database study was approved by the Intermountain Institutional Review Board (protocol code 10072305) with a waiver of consent. 

*Healthcare system and MI pathways:* Intermountain Health is a nonprofit, integrated healthcare system. At the time of this study, Intermountain Health included 24 hospitals and 215 clinics in Utah, Idaho, and Nevada. Intermountain Health’s electronic medical records (eMR) system has been operational for over 25 years. The study also accessed the complementary database containing catheterization laboratory records. To efficiently triage patients with MI to a hospital capable of emergent percutaneous coronary intervention (PCI), Intermountain Health has developed and implemented an acute coronary syndrome (ACS)-care pathway, which has achieved nation-leading outcome results. It also has an interest in the use of CAC scans to risk-stratify primary prevention patients for the selection of statin (and other) preventive medications [14].

*Study population and definitions:* Patients with either STEMI or NSTEMI presenting to Intermountain Health catheterization laboratories between 1 June 2001 and 31 January 2021, and who had a CAC scan within up to 2 years before or up to 2 years after this date, comprised the study population. This broad timeframe was chosen to optimize the numbers of No-SMRF MI patients with CAC scans, and with the understanding that CAC scores only change slowly over time and along the same percentile curves, and that they do not reverse with lipid-lowering or other preventive therapies. CAC scans were performed either as a stand-alone study (i.e., with a 64-slice computed tomography scanner) or as part of a positron emission tomography (PET)/CT stress test [15]. In a sensitivity analysis, we excluded patients whose CAC score was more than a week before or more than a week after the MI date.

SMRF was defined as a clinical diagnosis or treatment of hypertension, hyperlipidemia, diabetes, and/or being a current or former smoker. Diagnoses were ascertained from the Intermountain records database and catheterization laboratory records. In our healthcare system, hypercholesterolemia is defined as untreated total cholesterol ≥ 200 mg/dL, low-density-cholesterol (LDL-C) ≥ 130 mg/dL, or non-high-density-cholesterol (non-HDL-C) ≥ 130 mg/dL. Hypertension is defined as a confirmed systolic blood pressure ≥ 140 mmHg and/or diastolic pressure ≥ 90 mmHg. Diabetes is defined as a fasting glucose > 125 mg/dL or hemoglobin A1C ≥ 6.5%. 

*Study endpoints:* Endpoints included the proportion of No-SMRF patients who had CAC scores ≥ 50 percentile based on age and sex, determined using the MESA criteria [12]. The null hypothesis was that there would be a non-significant increase over expected (i.e., a score over the 50th population percentile). The co-primary hypothesis was that no more than 50% would have an indication for statin therapy, with or without aspirin, using national guidelines and our CorCal trial criteria (i.e., CAC score ≥ 100 AU and/or score ≥ 75 percentile) [13,14].

The primary short-term study outcome endpoint was a clinical major adverse cardiovascular event (MACE). Included in MACE were all-cause mortality, nonfatal MI, or a heart failure hospitalization within 60 days of the presenting MI. The primary long-term MACE outcome identified these events up to the end of follow-up, i.e., until 3 March 2021. Secondary endpoint events included individual event outcomes. 

*Statistical analysis:* Descriptive statistics were used to summarize demographics and clinical characteristics of patients enrolled in the study. After a normality check (Kolmogorov–Smirnov test) of continuous variables, the median and interquartile range (IQR) were reported, and count (%) was used to summarize categorical variables. Demographic and other baseline variables were then compared between SMRF and SMRF-less groups. Wilcoxon rank sum tests or chi-square tests were used to compare SMRF and SMRF-less groups for continuous variables (e.g., age, CAC score) or categorical variables (e.g., race, comorbidities), respectively. The primary end-point statistical analyses assessed the proportion of no-SMRF patients with CAC score ≥ 50 percentile and the proportion with ≥100 AU and/or ≥75 percentile. Testing for differences in CAC scores between SMRF categories used non-parametric Wilcoxon rank sum test. (Cox proportional hazard regression analysis was not conducted due to the small number of endpoint events.) Statistical analyses were performed with SAS version 9.4 (SAS Institute Inc., Cary, NC, USA). All *p*-values were 2-sided, and were evaluated at a significance level of 0.05.

## 3. Results

### 3.1. Patient Demographics

We identified 429 MI patients with a qualifying CAC scan, of which 60 had no SMRF and 369 had SMRF. The two groups were well matched for age (median 67 and 68 years, respectively), sex (63% and 61% male, respectively), most demographics, non-modifiable risk factors and interventions (Table 1). However, fewer patients without SMRF had a history of heart failure (8% vs. 21%, *p* = 0.02) or a family history of heart disease (8% vs. 21%, *p* = 0.02). At the time of MI, 35% (21/60) of No-SMRF patients with a CAC scan were on a statin, of which 10 were also taking aspirin.

### 3.2. CAC Score Findings

The qualifying CAC scan was performed at a median time from PET/CT scan to MI diagnosis of 0 days, and 40 of 60 patients had CAC scan within ±7 days of hospital admission/MI diagnosis. The timing of the 20 other scans was distributed throughout the ±2 years around the date of hospitalization, with a range of 678 days before to 667 days after the hospital admission/MI diagnosis date.

**Figure 1:**
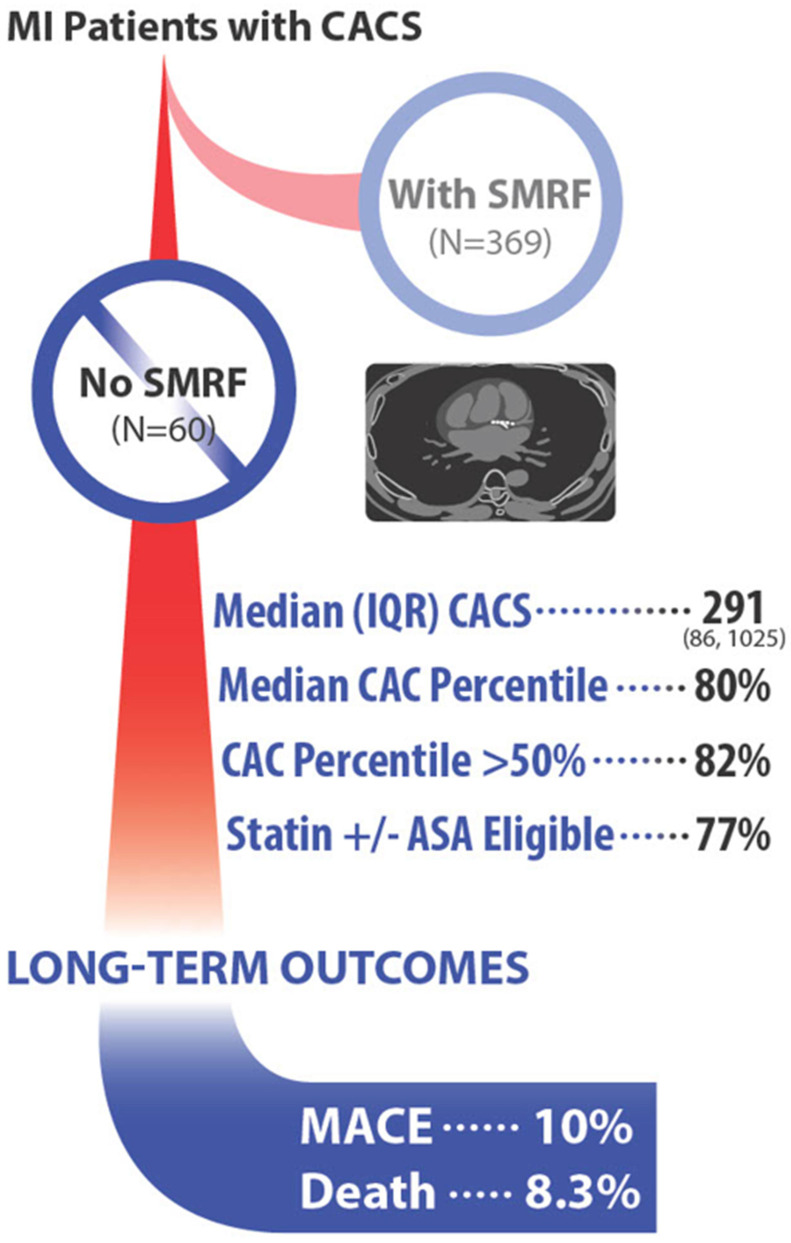
CAC scan results and outcomes in MI patients without SMRF. ASA = aspirin; CACS = coronary artery calcium scan; IQR = interquartile range; MACE = major adverse cardiovascular events; SMRF = standard modifiable risk factors. Outcomes were assessed at a mean of 2.2 years.

Patients with No-SMRF had a high prevalence and an increased percentile of CAC (Figure 1). In total, 82% had a CAC score more than or equal to the expected 50th percentile based on their age and sex. The median percentile was 80%, with an interquartile range of 55% to 85%, clearly exceeding the 50th percentile expected population age/sex average (Table 1). The median CAC score was 291 (IQR 85–1025), with a mean of 810 (range, 0–4402, SD 1489). A total of 77% met our criteria for statin primary ASCVD preventive therapy (with or without aspirin) (Table 1). (Given the absence of standard risk factors, few would have otherwise had an indication for therapy.) Patients with SMRF, as expected, had high CAC scores and percentiles (Table 1).

A sensitivity analysis (N = 40), excluding patients whose CAC scan was beyond one week before or after MI, found a similar or greater CAC burden than that of the full cohort, with median CAC score of 362 AU (IQR 130–1188), and a mean of 1021 (range, 0–4402), performed at a median time from MI diagnosis of 0 days.

### 3.3. Outcomes by CAC Status

In the absence of SMRF, outcomes tended to be more favorable, which became significant during long-term (mean, 2.2 y) follow-up (i.e., MACE 10% vs. 22%, *p* = 0.03) (Table 2). Confirming previous studies, lower CAC scores, regardless of SMRF status, were associated with lower MACE risk (Table 3). The long-term MACE rate was 10% when the CAC score was <100 AU and <75th percentile, compared to 22% for those with a CAC score ≥ 100 AU and/or ≥75th percentile. 

## 4. Discussion

*Summary of Study Findings:* To identify factors associated with an increased risk for MI in patients without SMRF, we tested CAC results in this system-wide retrospective observational database study. Despite absence of SMRF, we found that these patients presenting with an MI have a high prevalence and a high percentile of CAC, which would be an indication in almost 80% for the initiation of a statin [13,14] with or without aspirin therapy [16]. The 35% overall clinical statin use in the CAC scan cohort is less than one-half of this expectation, if based on foreknowledge of the CAC score. Statin use in treated patients was associated with various conditions (e.g., age-driven, high scores on the pooled cohort equation, knowledge of CAC from earlier scans, high normal standard risk factors, or other/unknown reasons).

We further compared No-SMRF patients to concurrently enrolled patients with SMRF and found, on average, calcium burden in No-SMRF patients to be lower and outcomes more favorable although still clinically important.

The findings of our prior reports on MI patients without SMRF suggest them to form an important part of MI presentations (i.e., up to 1 in 4 presentations), hence demanding of more attention as to how to identify them in advance and initiate preemptive preventive therapy. The findings of this study suggest that CAC scans represent a highly promising tool to augment assessment using standard risk factors; indeed, CAC may indicate preventive therapy in almost 80% of those patients destined otherwise to suffer an unexpected MI.

*Literature comparisons:* Over the past several years, interest has grown in patients presenting with MI but without SMRF. Reports from Australia, Europe and Asia have assessed the prevalence and prognosis of these patients [1,2,3,4,5,6,7,8,9,10]. These studies have reported that these No-SMRF MI patients are frequent (up to 27% of presentations). For STEMI patients, a worse in-hospital/short-term prognosis has been reported [2,3,4,5]. Our US healthcare studies identified No-SMRF status in 26% of STEMI presentations and 18% of NSTEMI presentations [6,10]. In contrast to several non-US reports, we observed adjusted early (<60 day) event rates that were not higher in No-SMRF patients with STEMI. Further, long-term outcomes were favorable, with reduced rates of MACE and hospitalizations for heart failure. Furthermore, after adjusting for lower use of guideline-indicated therapy, the high early mortality rates in non-US studies were attenuated [5]. For NSTEMI, frequency and prognosis in No-SMRF patients have been reported more variably, i.e., slightly less frequently than for STEMI and with a generally better prognosis [3,7,8]. However, the importance of NSTEMI is emphasized by the frequency of their presentations (i.e., 2–3 times that of STEMI) [3,6], and by their higher long-term mortality risk [7]. Indeed, most patients qualifying for the present study had NSTEMI, emphasizing their relevance to the present discussion. In our studies, we found a clear gradient of risk with the number of ICD-coded risk factor diagnoses, confirming their value in assessing cardiovascular risk [6,10]. 

A UK nationwide observational study assessed the frequency of SMRF-less status among NSTEMI patients. More than one-fifth had no SMRF [8]. Optimal guideline-recommended medications were given less often to SMRF-less than to SMRF patients. Outcomes (MACE and in-hospital mortality) were better for No-SMRF patients if propensity-matched. Our findings are similar in terms of the frequency of SMRF-less status and short-term prognosis, and we have added information on longer-term outcomes [10].

In a report from the SWEDEHEART Registry [9], 11% of NSTEMI patients were SMRF-less. As with the UK study, these patients received lower rates of guideline-recommended medications. Associated with this, SMRF-less patients had nominally higher rates of age- and sex-adjusted 30-day total and cardiovascular mortality. However, longer-term prognosis was better with No-SMRF status.

In an Asian study, STEMI patents without SMRF showed an increased short-term mortality risk. However, NSTEMI patients experienced no differences in mortality compared to patients with SMRF [3].

Quantitative CAC scans have been available for almost three decades [17]. Coronary plaque burden, as quantified by CAC, has been shown to be highly predictive of incident cardiovascular events [18,19,20]. Further, CAC scoring represents a risk assessment tool that is largely independent of equations using standard risk-factor assessment (such as the pooled cohort equation). Whereas risk factor assessment is probabilistic, CAC provides individual anatomic assessment of coronary plaque burden. Indeed, CAC scans have been able to reclassify more than 50% of patients at intermediate Framingham score risk to either a lower- or a higher-risk score [21]. The theoretical advantage of CAC scoring is that it is impacted not only by the six standard atherogenic risk factors, but also integrates all others, including unknown factors.

*Mechanistic considerations and non-standard risk factors:* CAC, although a powerful addition to risk assessment, is not perfect. Lipid rather than calcium content of plaques is believed to be requisite to rupture-prone plaques predisposing to MI [22]. A small percentage (1–2%) of symptomatic CAD is caused by non-calcified plaques, which are typically found in younger subjects (i.e., women < 55, men < 45 years old) [23]. Spontaneous coronary artery dissection (SCAD) is another cause of MI in middle-aged women that is not associated with calcium-marked coronary plaques [24]. These considerations could explain the less-than-perfect correlation of CAC and MI in patients without SMRF. Further, certain (e.g., spotty, speckled) features of coronary calcium have been reported to be more predictive of coronary-event-prone plaques [25], but this degree of fine detail is not routinely analyzed or reported in clinical CAC imaging reports.

Despite these limitations, standard CAC scores clearly add consistently, powerfully, and independently to coronary risk prediction in studied subject subsets. However, most studies and guidelines have focused on CAC utility only in populations graded as at intermediate risk based on standard risk factors [13]. Less recognized is the independent risk assessment provided by CAC scoring in patient subsets at the extremes of risk-factor determined risk. In our CorCal Vanguard study [14], we found a poor correlation between CAC and pooled cohort equation scores at the extremes of risk prediction. For example, more than one-third of subjects with a CAC sore of zero, for whom statin therapy generally is not recommended, had a potentially treatable pooled cohort equation-determined 10-year risk of ≥5%. Similarly, a report from the Multi-Ethnic Study of Atherosclerosis (MESA) found a poor correlation between the pooled cohort equation, which incorporates traditional risk factors, and CAC scores, even at the extremes of risk equation distribution. That is, a low CAC score often was seen with a high risk factor score and vice versa. The independent value of a high CAC burden was demonstrated by its association with an elevated risk, even in those without SMRF [26].

What, then, are candidate risk factors not covered by the six traditional ASCVD risk factors (i.e., age, sex, plus the four SMRF factors) that may contribute independently to the plaque burden? A first consideration is the sub-threshold elevations in the four SMRF. These factors are not always dichotomous but often incremental in their risk attributions. Subthreshold elevations in blood pressure, lipids, and glycemia likely represent unaccounted for coronary risk. Additional lifestyle factors should also be considered. The international INTERHEART case–control study found that adding five additional lifestyle factors to the standard risk factors could achieve up to a 94% prediction of population-attributable risk [27]. These included dietary patterns, physical activity, waist/hip ratio, psychosocial factors, and alcohol use. Environmental factors, e.g., air pollution, should be added to these expanded lifestyle factors in accounting for global coronary risk [28].

Biomarkers represent another potential addition to standard risk factors. In one study, the addition of N-terminal pro-brain natriuretic peptide, C-reactive protein, and troponin-I improved the 10-year MACE estimate when added to the traditional risk factors [29]. Novel lipid-related factors also are worthy of consideration. Specifically, lipoprotein(a) (Lp(a)), which is not measured in the standard lipid profile, is currently being highlighted as a common, but often missing, atherogenic risk factor [30,31,32]. Elevated Lp(a) is reported to be present in a fifth to a quarter of patients presenting with MI [32]. Lp(a) is highly heritable and unresponsive to diet and statin therapy. The proportion of No-SMRF MI patients with elevated Lp(a) is unknown but is clearly of interest. Fortunately, for those patients with elevated Lp(a), trials of potentially effective oligonucleotide-based therapies are underway.

Beyond rare familial hyperlipidemias, polygenetic contributions to coronary disease progression are being studied, including polygenic risk scores (PGRS). One study performed whole-genome sequencing in over 2000 multiethnic patients. A high polygenic risk score predicted more than a 3-fold increased odds of early-onset MI, similar to that among patients with monogenetic familial hypercholesterolemia. However, a high PGRS was 10-fold more frequent [33]. In a UK Biobank study, a PGRS (i.e., metaGRS) was developed based on 1.6 million variants. MetaGRS out-performed standard risk factor equations, with a hazard ratio of 4.2 (comparing top to bottom 20th percentile of metaGRS) [34].

As recently reviewed, mechanisms besides coronary plaques marked by CAC and non-standard risk factors also should be considered in SMRF-less MI subjects, especially in women [35]. These include depression as a risk factor and microvascular dysfunction and spontaneous coronary artery dissections (SCAD) as mechanisms.

*Clinical implications:* Our experience, and that of others, has recently emphasized the importance of the patient subset presenting with MI but without SMRF. These observations indicate gaps in our current primary CV risk algorithms. However, this study indicates that CAC scanning represents a promising tool to identify this additional at-risk population for preemptive primary preventive therapy in advance. Additional research is needed to identify the non-standard risk factors responsible for the emergence of atherosclerosis in these patients, which then can be separately targeted.

This study indicates that CAC, meanwhile, may integrate these additional but as yet undiscovered or unaccounted-for risk factors in determining coronary plaque burden and as a potentially superior way to assess coronary risk and guide the selection of currently available preventive therapies.

*Strengths and limitations:* This study and its companion studies [6,10] have the strength of using a longstanding, prospectively collected institutional eMR system, including integrated catheterization laboratory records. Intermountain also has a long-standing and efficient pathway for ACS management. A limitation of this study is that only a small percentage of MI patients had undergone CAC scans within a reasonable timeframe of their coronary event. A limitation of all observational study designs, such as this one, is the possibility of uncorrected selection biases that may impact outcome results. Also, risk factor accounting depended on ICD-coding of SMRF, which is known to have limitations in sensitivity and specificity. Some of our No-SMRF patients may have had borderline or isolated abnormal values of lipids or blood pressure, which may not have received a formal disease label in the electronic medical record. Our study’s racial and ethnic heritage was primarily White/Northern European. Results could differ when applied to different populations or to dissimilar healthcare systems. Some outpatient events may have occurred outside of Intermountain Health and may have been missed, although this likely would not favor one SMRF group over another.

## 5. Conclusions

Given the recent findings by us and others of a high prevalence of MI patients presenting without SMRF, we tested whether coronary artery calcium could represent a novel risk factor associated with MI in these patients. We found that, despite absence of standard risk factors, No-SMRF MI patients have a high prevalence and percentile of CAC scores (median, 80%), and nearly 80% would qualify for statin therapy, with or without aspirin. Wider application of CAC scans, to include those without SMRF, appears promising as a method to identify an additional at-risk population for consideration of primary preventive therapy.

## Figures and Tables

**Table 1:** Baseline characteristics of patients without and with SMRF with a CAC scan.

	No-SMRF (N = 60)		SMRF (N = 369)		
Age, median (IQR)	67 (59, 76)		68 (58, 75)		0.67
Gender, n (%)					0.73
Male	38	63.3%	225	61.0%	
Female	22	36.7%	144	39.0%	
Race, n (%)					0.84
White/Caucasian	57	95.0%	329	89.2%	
African American(Black)	1	1.67%	6	1.63%	
Asian	0	0%	8	2.17%	
Pacific Islander	0	0%	4	1.08%	
Unknown	2	3.33%	22	5.96%	
Family history of heart disease, n (%)	5	8.33%	76	20.6%	0.02
Comorbidities, n (%)					
Atrial Fibrillation (AF)	14	23.3%	96	26.0%	0.66
COPD	7	11.7%	52	14.1%	0.61
Depression	19	31.7%	116	31.4%	0.97
Heart Failure (HF)	5	8.33%	78	21.1%	0.02
Stroke	4	6.67%	20	5.2%	0.70
PCI performed, n (%)	28	46.7%	190	51.5%	0.49
CABG	9	15.0%	67	18.2%	0.55
STEMI	5	8.33%	19	5.2%	0.32
NSTEMI	55	91.7%	350	94.9%	
CAC score, median (IQR)	291 (86, 1025)		862 (225, 2156)		0.0003
CAC percentile, median (IQR)	80 (55, 85)		90 (75,95)		0.0002
CAC score >= 100 AU	44 (73.33%)		311 (84.28%)		0.04
CAC percentile >= 50	49 (81.67%)		334 (90.51%)		0.04
CAC percentile >= 75	35 (58.33%)		293 (79.40%)		0.0004
CAC score >= 100 AU or percentile >= 75	46 (76.67%)		321 (86.99%)		0.03

Analyses: Chi-squared tests for categorical variables and Wilcoxon rank sum tests for continuous variables were used to examine differences in baseline characteristics for patients by SMRF status. IQR = interquartile range, COPD = chronic obstructive pulmonary disease, AU = Agatston units.

**Table 2:** Outcomes by SMRF status.

	With SMRF		No-SMRF		
	n = 369 (86%)		n = 60 (14%)		
	n	%	n	%	*p*-Values
**60-day Outcomes**					
MACE	26	7.05%	3	5.00%	0.56
Death	17	4.61%	3	5.00%	0.89
MI	4	1.08%	0	0.00%	1.00
HF Admission	6	1.63%	0	0.00%	1.00
**Long-term (2.2** **±** **2.0 y) Outcomes**					
MACE	81	21.95%	6	10.00%	0.03
Death	53	14.36%	5	8.33%	0.21
MI	12	3.25%	0	0.00%	0.39
HF Admission	23	6.23%	1	1.67%	0.23

**Table 3:** Outcomes by CAC status: combined cohort.

	CAC ≥ 100 or Percentile ≥ 75		CAC < 100 & Percentile < 75		
	n = 367		n = 62		
	n	%	n	%	*p*-Values
Long-Term Outcomes					
MACE	81	22.07%	6	9.68%	0.03
Death	55	14.99%	3	4.84%	0.03
MI	10	2.72%	2	3.23%	0.69
HF Admission	22	5.99%	2	3.23%	0.55

No statistical interactions between SMRF, No-SMRF, CAC status, and outcomes. CAC in Agatston units.

## Data Availability

The data underlying this article cannot be shared publicly due to US privacy laws. Data are available upon reasonable request to the corresponding author.
